# Optimal Cutoff Age for Predicting Mortality Associated with Differentiated Thyroid Cancer

**DOI:** 10.1371/journal.pone.0130848

**Published:** 2015-06-23

**Authors:** Su-jin Kim, Jun Pyo Myong, Hyunsuk Suh, Kyu Eun Lee, Yeo-Kyu Youn

**Affiliations:** 1 Department of Surgery, Seoul National University Hospital & College of Medicine, Seoul, Korea; 2 Cancer Research Institute, Seoul National University College of Medicine, Seoul, Korea; 3 Division of Surgery, Thyroid Center, Seoul National University Cancer Hospital, Seoul, Korea; 4 Department of Occupational and Environmental Medicine, Seoul St. Mary's Hospital, College of Medicine, The Catholic University, Seoul, Korea; 5 Department of Surgery, Mount Sinai Beth Israel Hospital, Icahn School of Medicine at Mount Sinai, New York, United States of America; IPATIMUP/Faculty of Medicine of the University of Porto, PORTUGAL

## Abstract

Patient’s age at the time of diagnosis is an important prognostic factor for differentiated thyroid cancer (DTC) as reflected in various staging and risk stratification systems. However, discrepancies exist among the different staging systems on an optimal cut-off age for predicting the clinical outcome of patients with DTC. To determine the age at diagnosis most predictive of clinical outcomes of DTC, a population-based cohort study was performed composed of 35,323 patients with DTC between 1988 and 2010 using the Surveillance, Epidemiology, and End Results (SEER) database. The Youden index J was used to determine the most predictive age-at-diagnosis for thyroid-cancer-specific death. The multivariate Cox proportional hazards model was used to determine the hazard ratios (HRs) for each age group. With a median follow-up of 5.4 years (range, 0–22.9 years), DTC-associated mortality was 1.5% (n = 533) and the rate of death from overall cause was 7.0% (n = 2482). The optimal cutoff age at diagnosis for thyroid-cancer-specific death was 57. Multivariate analysis found that the age-at-diagnosis is the most prognostic factor for thyroid-cancer-specific death (HR 10.02, 95% CI 8.18–12.28). Age at diagnosis is the most important prognostic factor for DTC patients. Based on our analysis, age at diagnosis of 57 might be the optimal predictor of thyroid-cancer-specific death. This finding might be used as consideration in revision of the risk stratification system for treatment of DTC patients.

## Introduction

Thyroid cancer is the most common type of endocrine malignancy, and its incidence worldwide has rapidly increased during the last 3 decades [1, 2]. The majority of thyroid cancers are differentiated thyroid cancer (DTC), which is histologically subdivided into papillary thyroid cancer (80%–85%), and follicular thyroid cancer (10%–15%). Although most DTCs are indolent, with an excellent prognosis, some DTCs can spread, metastasize, recur, and eventually lead to death [1]. Therefore, risk-stratified treatment and surveillance are important for DTC management.

Unlike the other malignancies, patient’s age at the time of diagnosis is an important criterion as a prognostic determinant in most DTC staging systems [3–10]. However, significant variability exists among the systems. Certain staging systems use the age-at-diagnosis as a continuous variable [3–7] while other systems use it as a categorical variable with various cutoff ages [8–10]. Recent studies have evaluated the relationship between the age-at-diagnosis and the prognosis of patients with DTC, and attempted to establish the age that was most predictive of cancer-specific death [11,12]. However, because the study sample sizes were small, determining the optimal prognostic age were limited given the low incidence of DTC-related deaths.

Therefore, the aim of this study was to determine the age at the time of diagnosis that was most predictive of DTC-specific death using a population-based cohort. For the analysis, the Youden index *J* was utilized along with the Kaplan-Meier analysis, log-rank test, and multivariate analysis using the Cox proportional hazards model.

## Materials and Methods

### Ethics statement

The study design was approved by the Institutional Review Board of Seoul St. Mary’s Hospital, Korea (approval ID:KC14EISI0433). The dataset of SEER was openly accessed database. Cancer diagnoses are reportable diseases to the cancer registries (no consent required), including those that provide data to SEER. Therefore, the authors can access the processed publically available data from web site for SEER. Patients’ records were anonymized and de-identified prior to the analysis

### SEER database and eligible study population

The Surveillance, Epidemiology, and End Results (SEER) Program of the National Cancer institute (NCI) collects data on cancer incidence and survival from about 28% of the population of the United States. The SEER database includes demographic (age at diagnosis, gender, race), clinical (primary tumor site, tumor size and extent, operation type, lymph node involvement, distant metastasis), and prognostic (cancer-specific cause of death and overall cause of death) data. Ongoing quality control and quality improvement is performed by the SEER Program to ensure the collection of high-quality data. Additional details regarding the methods and design of the SEER Program, and the participating states have been reported elsewhere [1, 13]. The extent of tumor was defined as local (confined to the thyroid gland), regional (extension into adjacent tissue or lymph node involvement), or distant (metastatic). SEER data (Nov 2012 version) was used in this study [1].

**Fig 1:**
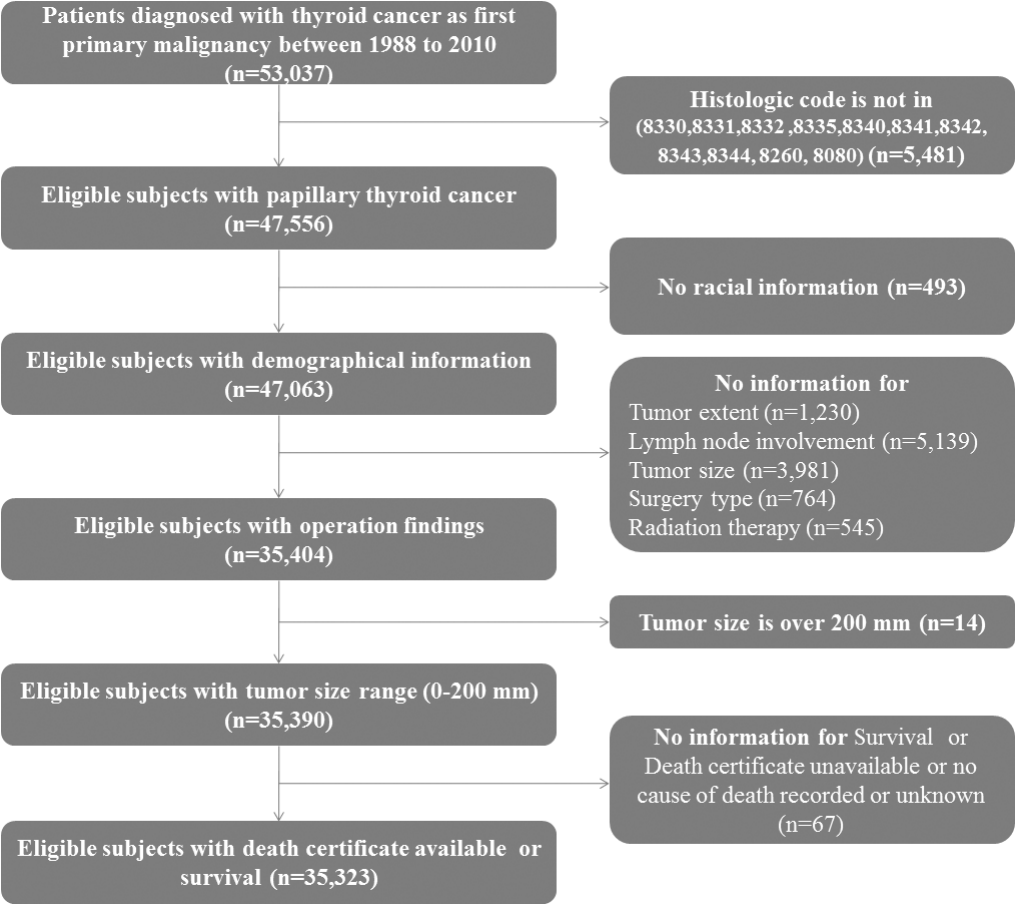
Flow chart of data collection.

The SEER program was contacted in March 2014 and the data from 1973–2010, representing 6,981,978 cancer cases were obtained. This study selected 53,037 patients from the SEER database who were diagnosed with thyroid cancer (C73.9) as the first primary malignancy from 1988 through 2010. Only the patients with histologically confirmed papillary or follicular thyroid cancer were included. Based on the *International Classification of Diseases for Oncology*, *Third Edition*, the following histologic subclasses were included for the study: 8340 (papillary carcinoma, follicular variant), 8341 (papillary microcarcinoma), 8342 (papillary carcinoma, oxyphilic cell), 8343 (papillary carcinoma, encapsulated), 8344 (papillary carcinoma, columnar cell), 8260 (papillary adenocarcinoma, not otherwise specified), 8080 (papillary carcinoma, not otherwise specified), 8330 follicular adenocarcinoma, not otherwise specified), 8331 (follicular adenocarcinoma, well differentiated), 8332 (follicular adenocarcinoma, trabecular), and 8335 (follicular carcinoma, minimally invasive). Patients with incomplete information on race, surgical findings (tumor size, extent, and lymph node involvement), adjuvant therapy, survival or cause of death were excluded. Another exclusion criteria was tumor measuring 200 mm or larger. The study cohort comprised of 35,323 patients with DTC. Fig 1 details numbers of inserted and excluded patients.

### Statistical analysis

**Fig 2:**
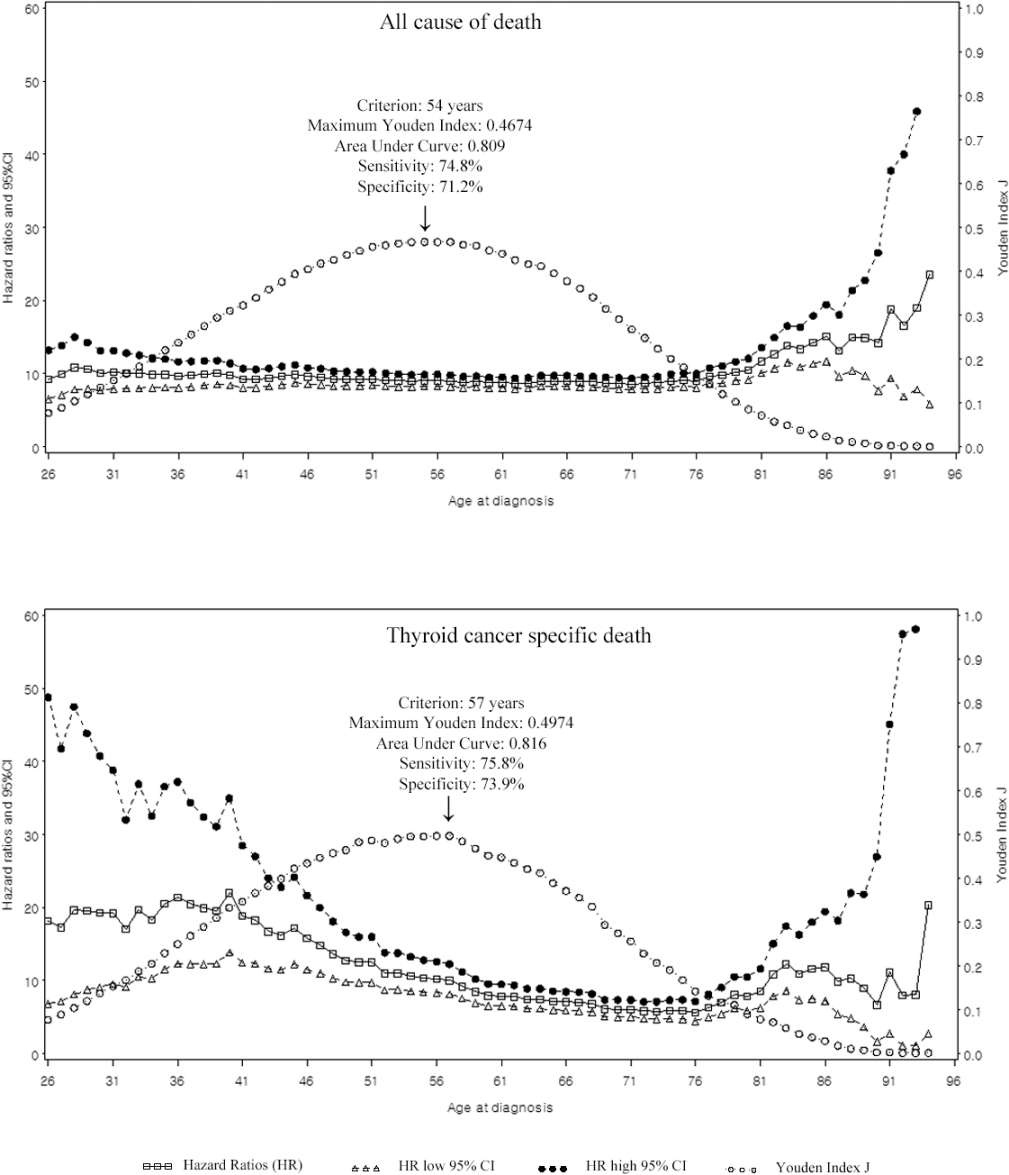
Multivariate Cox proportional hazard ratios for all cause of death, and cancer specific death with 95% confidence intervals, and Youden Index J derived from an univariate analysis at every age.

For descriptive statistics, the Student *t* test was employed for continuous variables while the chi-square test was employed for categorical variables. The Youden index *J* was used to determine the optimal cutoff age at the times of diagnosis for predicting overall and cancer-specific cause of death. The Youden index *J* was defined as: *J* = sensitivity + specificity-1 [14]. Youden index *J* in Fig 2 was derived from an univariate analysis. For the univariate assessments with dichotomous age as the predictive variable, survival curves for overall survival and cancer-specific survival were estimated using the Kaplan-Meier method and analyzed using the log-rank test. The dichotomous age points were chosen based on the Youden J index results in Fig 2.

After adjusting for gender, race, size of tumor, extent, extrathyroidal extension, lymph node metastasis, distant metastasis, extent of operation, and radiation therapy, proportional hazards model was used to determine the hazard ratios (HRs) corresponding to a dichotomized age as in the multivariable model. A two-sided *P*-value < 0.05 was considered statistically significant. Survival analysis was performed using the SAS version 9.2 software (Statistical Analysis Software Institute, Cary, NC, USA), and the Youden index *J* values were derived using the MedCalc Statistical Software version 13.1 (MedCalc Software bvba, Osten, Belgium).

## Results

Patients’ demographics and clinical characteristics are shown in Table 1. The majority of tumors were papillary thyroid cancer (93.1%). The mean age at diagnosis was 47.0 ± 15.3 years. The median follow-up period was 5.4 years (range, 0–22.9 years). During the follow-up period, overall mortality was 7.0% (n = 2,482), and thyroid cancer-specific mortality was 1.5% (n = 533).

**Table 1:** Demographics and clinical characteristics of patients with differentiated thyroid cancer (N = 35,323).

Characteristics	Number (%)
Age at diagnosis, years	
Mean±SD	47.0±15.3
Gender	
Male	7,934 (22.5)
Female	27,389 (77.5)
Race	
Black	1,920 (5.5)
White	29,261 (82.8)
Other	4,142 (11.7)
Histologic subtype	
Papillary	32,890 (93.1)
Follicular	2,433 (6.9)
Size of tumor, mm	
Mean±SD	19.3±16.3
Extent of tumor	
Localized	21,952 (62.2)
Regional	12,393 (35.1)
Distant*	978 (2.7)
Extrathyroidal extension	
Yes	5,479 (16.7)
No	27,411 (83.3)
Lymph node metastases	
Yes	8,410 (23.8)
No	26,913 (76.2)
Distant metastases	
Yes	978 (2.7)
No	34,345 (97.3)
Extent of operation	
Biopsy	244 (0.7)
Lobectomy	4,724 (13.4)
Subtotal or near-total thyroidectomy	2,699 (7.6)
Total thyroidectomy	27,655 (78.3)
Radiation therapy	
No	16,448 (46.6)
Radioactive I-131 ablation	17,630 (49.9)
External beam radiation therapy	1,245 (3.5)
Cause of death	
Death resulting from thyroid cancer	533 (1.5)
Death resulting from nonthyroid cancer	807 (2.3)
Death resulting from noncancer cause	1142 (3.2)
Median follow-up, years	5.42 (0–22.9)

*Extent of tumor: distant = distant metastases.

The maximum Youden Index *J* value was 0.4674 at 55 years for overall cause of death, and 0.4974 at 57 years for thyroid-cancer-specific death (Fig 2).

S1 Table summarizes the distribution of demographic and clinical characteristics according to the age-at-diagnosis cutoff values for overall and thyroid-cancer-specific survival (≤ 55, > 55; ≤ 57, > 57 years, respectively). Older participants were more likely to have larger tumors, lymph node metastases, and distant metastases than younger participants for both cutoff ages (all *P* < 0.001).

Kaplan-Meier survival function was evaluated for each age group for both overall survival and thyroid cancer specific survival (S1 Fig). The log-rank test demonstrated a statistically significant difference (*p* < 0.001) between both the overall survival curves, and the cancer specific survival curves based on the ages at diagnosis.

Tables 2 and 3 show the results of univariate and multivariate analysis for risk factors predictive of overall cause of death and thyroid-cancer-specific death. The cut-off ages were derived from univariate analysis (maximum Youden index *J* at OS and DSS). The age at diagnosis was the highest risk factor for both overall cause of death and thyroid-cancer-specific death by multivariate analysis (HR 9.04, 95% confidence interval [CI] 8.26–9.90; HR 10.02, 95% CI 8.18–12.28, respectively). For overall cause of death, age at diagnosis (> 55 years), male gender, race (black *vs*. white), extrathyroidal extension, lymph node metastasis, distant metastasis, extent of operation (biopsy, lobectomy *vs*. total thyroidectomy), radiation therapy (radioactive I-131 ablation, external beam radiation therapy *vs*. no radiation therapy) were significant predictive factors by multivariate analysis. For thyroid-cancer-specific death, age at diagnosis (> 57 years), male gender, size of tumor, extrathyroidal extension, lymph node metastasis, distant metastasis, extent of operation (total thyroidectomy), and postop radiation therapy were significant predictive factors based on the multivariate analysis.

**Table 2:** Univariate and multivariate analysis of factors predictive of overall survival of patients with differentiated thyroid cancer using Cox proportional hazards model.

	Univariate	Multivariate
Variable	Hazard ratio (95% CI)	Hazard ratio (95% CI)
`Age at diagnosis		
≤ 55 yr	Reference	Reference
> 55 yr	9.79 (8.96–10.70)	9.04 (8.26–9.90)
Gender		
Male	2.25 (2.07–2.44)	1.62 (1.49–1.76)
Female	Reference	
Race		
Black	1.33 (1.13–1.57)	1.27 (1.08–1.50)
White	Reference	Reference
Other	1.07 (0.95–1.20)	0.93 (0.83–1.05)
Size of tumor, mm		
≤10 mm	Reference	Reference
>10 mm	1.13 (1.03–1.23)	1.48 (0.95–1.16)
Extent of tumor		
Localized	Reference	Reference
Regional	1.52 (1.40–1.65)	1.21 (1.06–1.37)
Distant*	4.20 (3.64–4.84)	2.67 (2.20–3.24)
Extrathyroidal extension		
Yes	2.51 (2.31–2.73)	1.59 (1.42–1.78)
No	Reference	Reference
Lymph node metastases		
Yes	1.31 (1.20–1.43)	1.13 (1.01–1.27)
No	Reference	Reference
Distant metastases		
Yes	3.49 (3.05–4.00)	-*
No	Reference	-
Extent of operation		
Biopsy	2.65 (1.97–3.55)	2.76 (2.05–3.71)
Lobectomy	1.15 (1.03–1.28)	1.17 (1.04–1.32)
Subtotal or near total thyroidectomy	1.11 (0.99–1.26)	0.99 (0.88–1.12)
Total thyroidectomy	Reference	Reference
Radiation therapy		
No	Reference	Reference
Radioactive I-131 ablation	0.88 (0.81–0.95)	0.81 (0.74–0.89)
External beam radiation therapy	2.00 (1.73–2.32)	1.31 (1.12–1.53)

*Extent of tumor: distant = distant metastases

Note: CI, Confidence Interval.

**Table 3:** Univariate and multivariate analysis of factors predictive of cancer-specific survival of patients with differentiated thyroid cancer using Cox proportional hazards model.

	Univariate	Multivariate
Variables	Hazard ratio (95% CI)	Hazard ratio (95% CI)
Age at diagnosis		
≤ 57 yr	Reference	Reference
> 57 yr	11.81 (9.71–14.35)	10.02 (8.18–12.28)
Gender		
Male	2.46 (2.07–2.93)	1.32 (1.10–1.58)
Female	Reference	Reference
Race		
Black	0.91 (0.60–1.39)	0.97 (0.64–1.48)
White	Reference	Reference
Other	1.32 (1.04–1.67)	0.95 (0.70–1.13)
Size of tumor, mm		
≤10 mm	Reference	Reference
>10 mm	5.23 (3.82–7.17)	2.50 (1.80–3.46)
Extent of tumor		
Localized	Reference	Reference
Regional	7.37 (5.69–9.55)	2.39 (1.71–3.34)
Distant*	44.69 (33.58–59.47)	8.42 (5.70–12.44)
Extrathyroidal extension		
Yes	10.43 (8.72–12.48)	2.74 (2.20–3.42)
No	Reference	Reference
Lymph node metastases		
Yes	4.10 (3.45–4.86)	1.65 (1.34–2.02)
No	Reference	Reference
Distant metastases		
Yes	12.98 (10.72–15.72)	-*
No	Reference	-
Extent of operation		
Biopsy	2.76 (1.52–5.03)	5.46 (2.96–10.07)
Lobectomy	0.52 (0.38–0.72)	1.25 (0.90–1.75)
Subtotal or near total thyroidectomy	1.12 (0.87–1.46)	1.16 (0.89–1.50)
Total thyroidectomy	Reference	Reference
Radiation therapy		
No	Reference	Reference
Radioactive I-131 ablation	2.14 (1.74–2.63)	1.16 (0.93–1.45)
External beam radiation therapy	10.33 (8.03–13.27)	3.04 (2.33–3.97)

*Extent of tumor: distant = distant metastases

Note: CI, Confidence Interval.

Fig 2 shows the HR distribution of age at diagnosis and, its 95% CI, after adjusting for gender, race, size of tumor, extent, extrathyroidal extension, lymph node metastasis, distant metastasis, extent of operation, and radiation therapy. The Youden index *J* was also shown. The Youden index *J* and HR has similar a range of values (i.e. 50–60 years for OS and 50–58 for DSS). The cutoff value of 55 years for overall cause of death had a sensitivity and specificity of 74.8% and 71.2% (area under curve: 0.803), respectively. The cutoff value of 57 years for thyroid-specific-death had a sensitivity and specificity of 75.8% and 73.9% (area under curve: 0.816), respectively.

## Discussion

The purpose of this population-based cohort study was to evaluate age at the time of diagnosis and that might be most optimal for predictive the clinical outcome in DTC. A total of 35,323 patients with DTC from 1988–2010 were selected with clinicopathologic information from the SEER cancer database. For patients with DTC, age at diagnosis was the most important predictive factor for death from thyroid cancer (55 years: HR 10.02, 95% CI 8.18–12.28), and overall cause of death (57 years: HR 9.04, 95% CI 8.26–9.90) based on the multivariate analysis using the Cox proportional hazards models.

Prognosis of majority of DTCs are excellent, however, about 10% of patients eventually die of the disease [15–17]. Therefore, a number of studies have tried to establish a risk stratification system or staging system to classify high-risk and low-risk patients based on demographic, and clinicopathologic factors. In DTC, age at diagnosis is considered to be a strong prognostic factor and, therefore, it is integrated in the majority of staging systems [3–10,18,19]. The tumor, node, metastases (TNM) staging system [3] developed jointly by the Union International Contre le Cancer (UICC) and the American Joint Committee on Cancer (AJCC), classifies DTC patients using a cutoff value of 45 years for age at diagnosis. Other staging systems such as Age, Metastases, Extent, Size (AMES) [4]; Grade, Age, Metastases, Extent, Size (GAMES) [5]; Noguchi et al. [6]; and the National Thyroid Cancer Treatment Cooperative Study (NTCTCS) [7] have adopted a similar age-based staging system using diverse cutoff values for age to risk-stratify patients with DTC. In contrast, the systems used by the following: European Organization for Research and Treatment of Cancer (EORTC) [8]; Age, Grade, Extent, Size (AGES) [9]; and Metastasis, Age, Completeness of Resection, Invasion, Size (MACIS) [10] systems have adopted the age factor as a continuous variable for calculating prognostic scores. While age is generally considered as an important determinant of clinical outcome for DTC, an optimal cutoff age has not been well established. The present study tried to find an optimal cutoff point with Youden index *J* which will reflect the maximum area under curve (AUC). The specific ages (age at 55 for OS, and 57 for DSS) were likely to have maximum Youden index *J*, however, it was derived with univariate results. Therefore, further prospective and well-designed research for suggesting proper age cut-off levels should be followed.

Interestingly, these finding are similar to those of recent studies [11,20]. Jonklass et al [11] suggested a cutoff value based on the age when most women attained menopause [21,22], and hypothesized that the age of 55 was appropriate for DTC staging systems using the Cox proportional hazard model after adjusting for age at diagnosis, gender, and TNM stage. Mazurat [20] divided study group into small age groups to find optimal cutoff value of age and also suggested that 55 years is better than 45 years for risk stratification in DTC patients using multivariate Cox proportional hazard model. Therefore, results of our study could be useful for redefining the existing staging systems for DTC. Nevertheless, controversies exist with advocates for both increased and decreased cut-off age for prognosis. Of note, Tran Cao et al [23] found that starting at age 30, each age decade was independently associated with a worse prognosis. Furthermore, Bischoff et al [24] found that 5-year survival decreased in each age category with 5-year increment without an inflection point at age 45 for papillary thyroid cancer.

Recently, using the SEER database, Yang et al. [13] analyzed 29,225 patients with thyroid cancers of all subtypes, and developed a nomogram based on a competing risk model to predict probability of death in thyroid cancer patients. Since most DTC patients have an indolent course with a low mortality rate, analyzing the probability of cancer-specific death can be challenging. Unlike the single-institution-based study [12], the population-based SEER cancer database has allowed simultaneous examination of a number of prognostic factors in a large group of patients who were not subjected to selection or referral biases [18]. A population-based cohort study can depict more reliable, and generally applicable information [13].

Therefore, we believe that this study provides generally acceptable information for healthcare providers and patients, regarding the effect of age at the time of diagnosis and DTC. The results of this study would be helpful to identify at-risk patients for DTC and guide treatment plans, including the extent of operative management as well as postoperative radioactive iodine therapy, and follow-up.

Although we analyzed the population-based cohort and used multivariate analyses to account for confounding factors, this study has some limitations. We could not determine the risk factors for DTC recurrence using the SEER database, or determine whether the age at diagnosis is also an important predictor of DTC recurrence. Because the SEER database focuses on gathering reliable information during the diagnostic period often without a mortality data, little information were collected on later events [25]. All models are main effect models, therefore, our method of Youden index *J* analysis may not have accounted for the other prognostic factors related to age. In addition, we could not confirm that the cutoff age of 57 for DTC-specific death was a better predictor than the cutoff age of 45 used in the TNM staging system. This is due to the fact that the SEER database prior to 2004 lacks, any information on thyroid cancer TNM staging including the lymph node metastasis status such as central lymph node metastases (N1a) vs.lateral lymph node metastases (N1b).

## Conclusions

In conclusion, this study found that the age-at-diagnosis is the most important prognostic factor for patients with DTC and the age cutoff value of 57 years may better risk stratify and predict the cancer-specific death for DTC patients. This finding might be used as consideration in revision of the risk stratification system for treatment of DTC patients.

## Supporting Information

S1 Fig Survival curve for overall survival and thyroid cancer specific survival by age cut-off levels. (TIF)

S1 Table Demographic and clinical characteristics of patients with differentiated thyroid cancer according to cutoff ages at diagnosis. (DOCX)
