# Supplementary figures and images for: Optimal Cutoff Age for Predicting Mortality Associated with Differentiated Thyroid Cancer
Source: PLoS One. 2015 Jun 23;10(6):e0130848. doi: 10.1371/journal.pone.0130848 (PMC4477980; doi:10.1371/journal.pone.0130848)

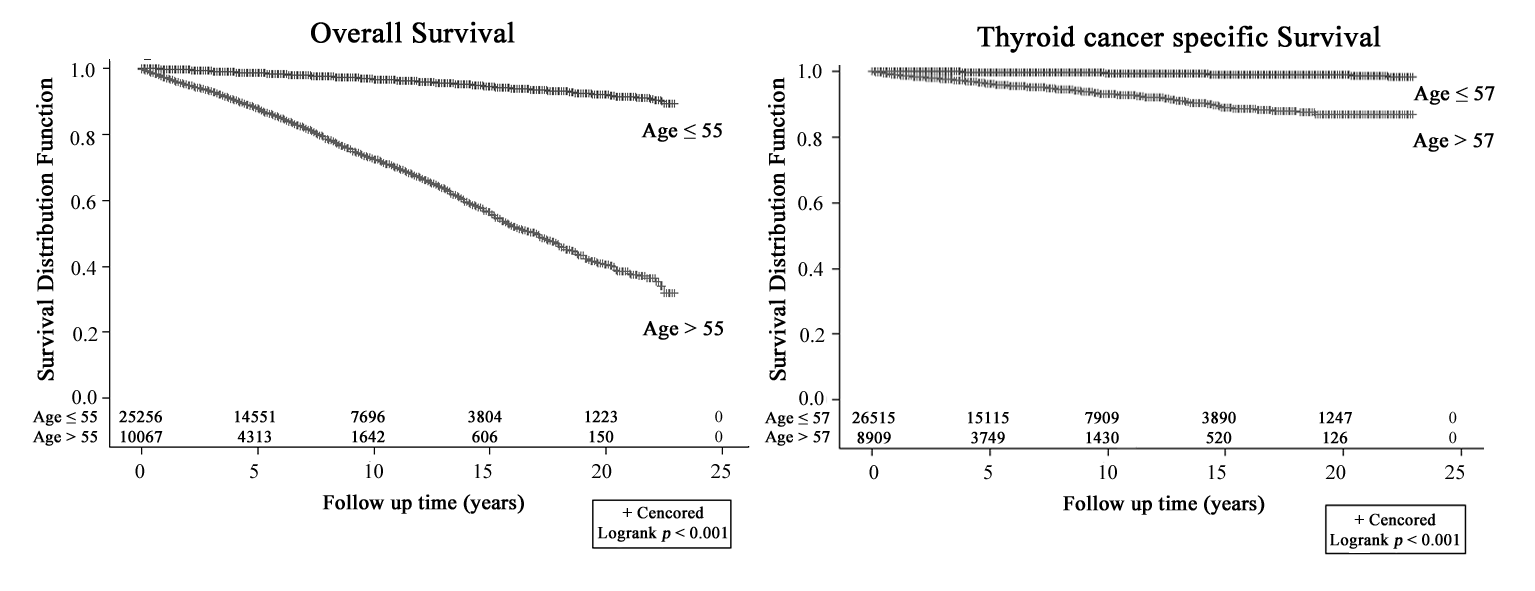

Supplement: S1 Fig — (TIF) [file pone.0130848.s001.tif]
