# Supplementary material for: Optimal Cutoff Age for Predicting Mortality Associated with Differentiated Thyroid Cancer
Source: PLoS One. 2015 Jun 23;10(6):e0130848. doi: 10.1371/journal.pone.0130848 (PMC4477980; doi:10.1371/journal.pone.0130848)
Supplement: S1 Table — (DOCX) [file pone.0130848.s002.docx]

**S1 Table. Demographic and clinical characteristics of patients with differentiated thyroid cancer according to cutoff ages at diagnosis**

|  | Overall survival | | | Thyroid-cancer-specific survival | | |
| --- | --- | --- | --- | --- | --- | --- |
| Characteristics | Age ≤ 55 | Age > 55 | *P*-value | Age ≤ 57 | Age > 57 | *P*-value |
| Age at diagnosis, years  Mean±SD | 40.2±10.4 | 67.5±7.4 | <0.001 | 39.4±9.9 | 66.1± 7.8 | <0.001 |
| Gender  Male  Female | 4,943 (19.6)  20,313 (80.4) | 2,991 (29.7)  7,076 (70.3) | <0.001 | 5,301 (20.0)  21,214 (80.0) | 2,633 (29.9)  6,175 (70.1) | <0.001 |
| Race |  |  | 0.591 |  |  | 0.288 |
| Black | 1,355 (5.4) | 565 (5.6) |  | 1,447 (5.4) | 473 (5.4) |  |
| White | 20,972 (83.0) | 8,289 (82.3) |  | 21,985 (83.0) | 7,276 (82.6) |  |
| Other | 2,929 (11.6) | 1,213 (12.1) |  | 3,083 (11.6) | 1,059 (12.0) |  |
| Histologic subtype |  |  | <0.001 |  |  | <0.001 |
| Papillary | 23,645 (93.6) | 9,245 (91.8) |  | 24,831 (93.6) | 8,059 (91.5) |  |
| Follicular | 1,611 (6.4) | 822 (8.2) |  | 1,684 (6.4) | 749 (8.5) |  |
| Size of tumor  Mean±SD (mm) | 19.0±15.3 | 20.3±19.1 | <0.001 | 19.1±15.3 | 19.9±18.7 | <0.001 |
| ≤10 mm | 16,836 (66.7) | 6,125 (60.8) | <0.001 | 17,558 (66.2) | 5,403 (61.3) | <0.001 |
| >10 mm | 8,420 (33.3) | 3,942 (39.2) |  | 8,957 (33.8) | 3,405 (38.7) |  |
| Extent of tumor |  |  | <0.001 |  |  | <0.001 |
| Localized | 15,517 (61.4) | 6,435 (63.9) |  | 16,380 (61.8) | 5,572 (63.3) |  |
| Regional | 9,142(36.2) | 3,251 (32.3) |  | 9,512 (35.9) | 2,881 (33.7) |  |
| Distant* | 597 (2.4) | 381 (3.8) |  | 632 (2.4) | 355 (4.0) |  |
| Extrathyroidal extension |  |  | <0.001 |  |  | <0.001 |
| Yes | 3,701 (14.7) | 2,053 (20.4) |  | 3,913 (14.8) | 1,841 (20.9) |  |
| No | 21,555 (85.3) | 8,014 (79.6) |  | 22,602 (85.2) | 6,967 (79.1) |  |
| Lymph node metastases |  |  | <0.001 |  |  | <0.001 |
| Yes | 6,600 (26.1) | 1,810 (18.0) |  | 6,826 (25.7) | 1,584 (18.0) |  |
| No | 18,656 (73.9) | 8,257 (82.0) |  | 19,689 (74.3) | 7,224 (82.0) |  |
| Distant metastases |  |  | <0.001 |  |  | <0.001 |
| Yes | 597 (2.4) | 381 (3.8) |  | 632 (2.4) | 355 (4.0) |  |
| No | 24,659 (97.6) | 9,686 (96.2) |  | 25,892 (97.6) | 8,453 (96.0) |  |
| Extent of operation |  |  | <0.001 |  |  | <0.001 |
| Biopsy | 143 (0.6) | 101 (1.0) |  | 147 (0.6) | 97 (1.1) |  |
| Lobectomy | 3,170 (12.5) | 1,554 (15.4) |  | 3,336 (12.6) | 1,388 (15.8) |  |
| Subtotal or near-total thyroidectomy | 1,914 (7.6) | 785 (7.8) |  | 2,002 (7.5) | 697 (7.9) |  |
| Total thyroidectomy | 20,028 (79.3) | 7,627 (75.8) |  | 21,029 (79.3) | 6,626 (75.2) |  |
| Radiation therapy |  |  | <0.001 |  |  | <0.001 |
| No | 11,293 (44.7) | 5,155 (51.2) |  | 11,905 (44.9) | 4,543 (51.6) |  |
| Radioactive I-131 ablation | 13,124 (52.0) | 4,506 (44.8) |  | 13,733 (51.8) | 3,897 (44.2) |  |
| External beam radiation therapy | 839 (3.3) | 406 (4.0) |  | 877 (3.3) | 368 (4.2) |  |
| Median follow-up, years | 6.1 (0-22.9) | 4.2 (0-22.9) |  | 6.0 (0-22.9) | 4.2 (0-22.9) |  |
| Death resulting from thyroid cancer |  |  |  |  |  |  |
| No |  |  |  | 26,376 (99.5) | 8,414 (95.5) | <0.001 |
| Yes |  |  |  | 139 (0.5) | 394 (4.5) |  |
| Death resulting from over-all cause |  |  | <0.001 |  |  |  |
| No | 24,560 (97.2) | 8,281 (82.3) |  |  |  |  |
| Yes | 696 (2.8) | 1,786 (17.7) |  |  |  |  |
| Total | 25,256 (100.0) | 10,067 (100.0) |  | 26,515 (100.0) | 8,808 (100.0) |  |

*Extent of tumor: distant = distant metastases
